# Effects of suppressing gonadal hormones on response to novel objects in adolescent rats

**DOI:** 10.1016/j.yhbeh.2011.08.015

**Published:** 2011-11

**Authors:** De-Laine M. Cyrenne, Gillian R. Brown

**Affiliations:** School of Psychology, University of St Andrews, South Street, St Andrews, KY16 9JP, UK

**Keywords:** Sex difference, Novelty, Novel object recognition, Testosterone, Estradiol, Antide

## Abstract

Human adolescents exhibit higher levels of novelty-seeking behaviour than younger or older individuals, and novelty-seeking is higher in males than females from adolescence onwards. Gonadal hormones, such as testosterone and estradiol, have been suggested to underlie age and sex difference in response to novelty; however, empirical evidence in support of this hypothesis is limited. Here, we investigated whether suppressing gonadal hormone levels during adolescence affects response to novelty in laboratory rats. Previously, we have shown that male adolescent Lister-hooded rats (postnatal day, *pnd*, 40) exhibit a stronger preference than same-aged females for a novel object compared to a familiar object. In the current study, 24 male and 24 female Lister-hooded rats were administered with Antide (a gonadotrophin-releasing hormone antagonist), or with a control vehicle solution, at pnd 28. Antide provided long-term suppression of gonadal hormone production, as confirmed by ELISA assays and measurement of internal organs. Response to novel objects was tested at pnd 40 in Antide-treated and control subjects using a ‘novel object recognition’ task with a short (2-minute) inter-trial interval. In support of previous findings, control males exhibited a stronger preference than control females for novelty when presented with a choice of objects. Antide-treated males exhibited a significantly lower preference for novel objects compared to control males, whilst Antide-treated females did not differ significantly from control females in their preference for novelty. Antide treatment did not affect total time spent interacting with objects. We discuss how gonadal hormones might influence sex differences in preference for novelty during adolescence.

## Highlights

► Adolescent male rats exhibited a stronger preference for novel objects than adolescent females. ► Suppressing gonadal hormones in adolescent males reduced preference for novel objects. ► Suppressing gonadal hormones in adolescent females did not affect response to novelty. ► The effect in males could result from interactions between testosterone and the dopamine system. ► The study has potential implications for understanding adolescent susceptibility to drug abuse.

## Introduction

In human beings, adolescents generally exhibit higher levels of novelty-seeking behaviour than younger and older individuals (Arnett, 1992; Kelley et al., 2004), and males report engaging in more novelty-seeking behaviour than females from adolescence onwards (Zuckerman, 2006). Researchers have argued that these age and sex differences in behaviour could be adaptive; for instance, adolescents potentially gain important information about their environment by seeking out novel experiences at a time when they are becoming independent from their parents (Chambers et al., 2003), and sexual selection pressures can favour riskier behavioural strategies in males than females (Daly and Wilson, 1983; Spear, 2000). Thus, certain groups of individuals, particularly adolescent males, might be more strongly predisposed than other groups to prefer novel stimuli.

Understanding the mechanisms that might predispose adolescent males towards a preference for novelty is important, given that novelty-seeking has been closely linked to drug abuse (Bardo et al., 1996; Roberti, 2004) and adolescence is a period of significant vulnerability to addiction (Chambers et al., 2003; Crews et al., 2007; Spear, 2000; Witt, 2007). Gonadal hormones, such as testosterone and estradiol, have been suggested to play a role in the expression of novelty-seeking behaviour during adolescence and the difference in novelty-seeking tendencies between males and females (Doremus-Fitzwater et al., 2010; Ernst et al., 2009; Forbes and Dahl, 2010; Kuhn et al., 2010). However, experimental evidence in support of this hypothesis is currently limited. The aim of this study was to examine the effects of suppressing gonadal hormone production during adolescence on response to novelty in male and female laboratory rats (*Rattus norvegicus*).

Adolescence in rats encompasses the period from weaning (postnatal day, *pnd*, 21) to early adulthood (pnd 60), and this period can be further divided into early adolescence (pnd 21–33), mid-adolescence (pnd 34–46) and late adolescence (pnd 47–59) (based on Tirelli et al., 2003). During early adolescence, circulating ovarian hormone levels begin to rise in female rats and ovarian weight increases, and, similarly, testosterone levels rise and testicular weight increases in males (Gabriel et al., 1992; Pignatelli et al., 2006). In the wild, these young animals begin to explore the area immediately outside of the natal burrow (Calhoun, 1963). During mid-adolescence, females exhibit vaginal opening and irregular ovarian cycling, whilst testosterone levels continue to rise in males (Gabriel et al., 1992), and wild rats follow the mother on foraging trips away from the burrow at this age (Calhoun, 1963). By late adolescence, females exhibit regular ovarian cycles, and males are capable of producing fertile sperm (Gabriel et al., 1992; Tentler et al., 1997), and late adolescents often sleep in nest chambers away from the mother and littermates (Calhoun, 1963). After this age, young adult rats engage in sexual and aggressive interactions with other individuals and disperse from the natal area (Calhoun, 1963). As in most rodent species, male rats typically move further away from the natal burrow system than females (Calhoun, 1963; Krebs et al., 2007).

Previously, we have shown that mid-adolescent male rats (pnd 40) exhibit a stronger preference than same-aged females for a novel object compared to a familiar object in a laboratory setting (Cyrenne and Brown, 2011), and this sex difference is not observed in younger (pnd 28) or older (pnd 80) age groups (Cyrenne and Brown, 2011). To examine response to novelty, we have used a variant of the ‘novel object recognition’ (NOR) task (Berlyne, 1950; Ennaceur and Delacour, 1988), which forces rodents to *confront* novelty and also provides subjects with the opportunity to *choose* between a novel and a familiar stimulus. The procedure is to familiarise an animal to a novel arena, then place two objects into the arena and allow the animal to interact with the objects. During this first trial, Trial 1, the subject is confronted with novelty. One of the objects is then replaced with a new item and, in Trial 2, the animal has the choice of interacting with the novel or the familiar object. Previous studies have shown that rodents generally spend more time interacting with the novel than the familiar object in Trial 2 (Dere et al., 2007; Ennaceur and Delacour, 1988). The NOR task has been used extensively in rodent memory research, and increasing the delay between the first and second trials to several hours reduces the difference in response to the novel and familiar objects (e.g. 4 h or 24 h: Ennaceur and Delacour, 1988; Şik et al., 2003). However, the NOR task also allows researchers to investigate the mechanisms involved in novelty preference (Besheer et al., 2001), and we used a short interval between the two trials (i.e. 2 min) to reduce the probability that differences in response to the objects between groups would result from differences in memory ability.

The current study investigated whether the sex difference in performance on the NOR task during mid-adolescence is influenced by suppression of circulating gonadal hormone levels. To suppress gonadal hormone production from early adolescence onwards, male and female rats were administered with a long-acting gonadotrophin-releasing hormone (GnRH) antagonist, Antide. GnRH antagonists act at the pituitary gland, where they strongly bind with GnRH receptors and hence prevent endogenous GnRH from stimulating gonadotrophin production (reviewed by Herbst, 2003). As a consequence of the lack of gonadotrophins, gonadal hormone production is blocked and gonadal development is retarded. The effects of these drugs are rapid in onset, and, unlike traditional gonadectomy, surgery is not required, as antagonists are administered via subcutaneous or intra-peritoneal routes. In our study, Antide was administered subcutaneously at pnd 28, which marks the start of the rise in circulating gonadal hormone levels in both sexes (Gabriel et al., 1992; Pignatelli et al., 2006), and the chosen dose was predicted to suppress hormone production through to pnd 40 in both males and females (Habenicht et al., 1990; Takeyoshi et al., 2002). We used a GnRH antagonist, rather than gonadectomy, as the effects of GnRH antagonists wear off over time and future studies could potentially investigate the effects of ‘delaying’ puberty.

The response of Antide-treated and control subjects to novelty was tested in the NOR task at pnd 40 using a 2-minute inter-trial interval. In addition to measuring the strength preference for the novel object in Trial 2, time spent moving and total amount of time spent in contact with the objects during both trials was recorded, in order to examine whether the hormone manipulations also influenced these measures. We hypothesised that control males would exhibit a stronger preference for the novel object than control females at pnd 40 (Cyrenne and Brown, 2011), and that Antide treatment would influence response to novelty in one or both sexes. A small number of studies have suggested that preference for novel objects is reduced by removal of gonadal hormones in adult rodents (e.g. males: Aubele et al., 2008; Ceccarelli et al., 2001; females: Wallace et al., 2006). However, these studies used a NOR task with longer inter-trial intervals than the current study; thus, the effects of gonadectomy might have resulted from changes in memory performance rather than from changes in initial preference for novelty. Whether manipulating gonadal hormone levels influences preference for novelty at short inter-trial intervals in adolescent rats has not been examined previously.

## Methods

### Subjects and housing

The subjects were 24 male and 24 female Lister-hooded rats bred in-house from stock (Harlan, U.K.). All animals were housed in cages (measuring 25 cm × 45 cm × 15 cm) with ad libitum access to soy-free rodent pellets and water. Housing rooms were controlled for temperature (20 ± 1 °C) and humidity (55 ± 5%), and maintained on a 12-hour light:dark cycle (lights on 7 am). From pnd 17, pups were handled once per day and were weaned into same-sex sibling groups at pnd 21, then housed as same-sex pairs from pnd 28 onwards. The subjects were taken from 16 litters, with no more than one individual in each experimental group taken from a single litter. All appropriate guidelines and regulations were adhered to, as set out in the Principles of Laboratory Animal Care (NIH, Publication No. 85–23, revised 1985) and the UK Home Office Animals (Scientific Procedures) Act 1986.

### Experimental design

On pnd 28, experimental animals (12 males, 12 females) were treated with a gonadotrophin-hormone releasing hormone (GnRH) antagonist, Antide (Bachem Distribution Services, Germany; dissolved in 1:1 mixture of propylene glycol:saline) via subcutaneous injection at a dose of 6 mg/kg (based on Habenicht et al., 1990; Takeyoshi et al., 2002). Control animals (12 males, 12 females: cage-mates of Antide-treated subjects) were administered with a subcutaneous injection of the vehicle solution at pnd 28. For all subjects, body weight and ano-genital distance were measured at pnd 21, 28, 35 and 40. Behavioural testing was conducted on pnd 40. Immediately after testing, subjects were euthanised, and testes and uteri were removed and weighed. Blood was also collected for hormonal analysis at this time, and the serum was stored at − 80 °C prior to assay.

### Hormone assays

Serum samples from male subjects were analysed using a testosterone ELISA assay kit (Assay Designs, Enzo Life Sciences, U.K.). Samples were diluted (1:10) and run in duplicate. This kit has a lower limit of detection of 5.67 pg/ml, an inter-assay coefficient of variation of 11.3% and an intra-assay coefficient of variation of 10.0%. Serum samples from female subjects were analysed using a progesterone ELISA assay kit (Assay Designs, Enzo Life Sciences, U.K.). Samples were diluted (1:100) and run in duplicate. This kit has a lower limit of detection of 8.57 pg/ml, an inter-assay coefficient of variation of 8.3% and an intra-assay coefficient of variation of 5.4%.

### Apparatus

The apparatus for the NOR task was a wooden, light grey-painted square chamber (67 cm × 67 cm × 45 cm, l × w × h: similar in size to previous studies, e.g. Cain et al., 2005; Ennaceur and Delacour, 1988; Inagaki et al., 2010). Five objects (yellow rubber toy, glass jar filled with rocks, blue plastic bottle filled with sand, orange plastic toy watering can, multi-coloured LEGO® Duplo blocks tower) of similar size (approximately 15 cm high × 6 cm diameter) were used in the experiment. Objects were chosen that would deter climbing and chewing. A pilot study with male and female adult rats showed that, from a range of objects, the amount of time spent interacting was very similar for all of these items. The chamber was surrounded by a black curtain, and a video camera attached to the ceiling relayed images to a computer. All tests were conducted between 09:00 and 14:00 h in the same testing room under dim, white light (approximately 25 lx), and a white noise generator was used to mask external sounds.

### Behavioural testing

**Fig. 1:**
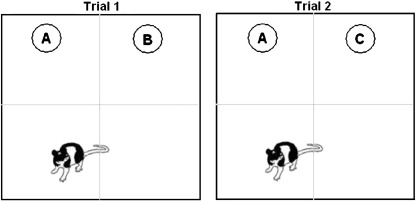
Apparatus for behavioural testing, where A, B and C represent the objects.

At the beginning of a session, a subject was brought to the testing room in a carrying box (42 cm × 26 cm × 13 cm, l × w × h) and placed into the empty chamber for a 10-minute familiarisation period. The animal was then returned to the carrying box for 2 min whilst the chamber was cleaned with a 70% ethanol solution and allowed to air dry. Two objects were placed into the chamber in adjacent quadrants (Fig. 1), and the animal was placed into an empty quadrant, facing away from the objects. During this trial, Trial 1 (5 min, as in previous studies, e.g. Frick and Gresack, 2003; Silvers et al., 2007), the subject had the opportunity to interact with the two objects. The animal was then placed back into the carrying box for an inter-trial interval of 2 min, during which the chamber and objects were cleaned with the ethanol solution and one of the objects was replaced by a novel object. The animal was then reintroduced to the chamber for Trial 2 (5 min). The object that remained from the first trial was considered the familiar object, and the new object was considered the novel object. At the end of Trial 2, the subject was immediately returned to the home cage, and the chamber and all objects were cleaned with the ethanol solution. Pilot studies in our laboratory confirmed that adult rats perform similarly on the NOR task when either two identical objects or two different objects are used during training (Cyrenne and Brown, unpublished). The objects used were counterbalanced across subjects and between treatment groups, and whether the left-hand or right-hand object was replaced in Trial 2 was also counterbalanced.

### Behavioural measures

All sessions were analysed using EthoVision XT 5.0 software (Noldus Information Technology, Netherlands, 2008). During Trials 1 and 2, the software recorded the *amount of time spent moving* by the subject. By delineating an area around each object (an additional 2 cm beyond the object) and by tracking the position of the animal's nose, the software was also able to calculate the *time spent interacting with each object* during Trials 1 and 2 (walking past the object, backing into an object and tail-only contact were thus excluded). We confirmed that the EthoVision measure of time spent interacting with an object strongly correlated with data collected by a human observer (*r*_99_ = .74, *p* < .001), and previous studies have also shown a strong correlation between automated and human-observer measures of novel object interactions in arenas of various sizes (Rutten et al., 2008; Silvers et al., 2007).

Time spent with the novel and familiar objects in Trial 2 was converted to a measure of *preference for novelty*, calculated as the proportion of time spent interacting with the novel versus the familiar object in Trial 2, converted to a percentage [(Time with novel – Time with familiar)/(Time with novel + Time with familiar)] × 100. A positive value indicates a preference for the novel object, whilst a negative value indicates a preference for the familiar object, and a score of zero indicates equal preference for the two objects. Any animal that did not exhibit a minimum of 5 s of total contact with the objects in Trial 1, and at least 1 second contact with either object in Trial 2, was excluded from the study; no animals were removed based on these criteria.

### Statistical analyses

T-tests and repeated-measures analyses of variance (ANOVAs) were used to analyse hormone data and physical measurements. Repeated-measures ANOVAs were used to examine behavioural data, with *sex* and *treatment group* as independent variables and *trial* as the repeated measure. Three-way interactions are only reported when significant. One-sample t-tests were used to examine whether subjects showed a significant preference for the novel object in Trial 2 (preference values were compared to zero, indicating no preference). Pearson's correlations were used to examine relationships between the behavioural measures and, where significant, analyses of co-variance were carried out. To examine whether individual responses to objects influenced preference scores, object identity was included as a random factor in the analyses. An α value of .05 was used throughout, and Bonferroni pairwise comparisons were used where appropriate. Analyses were conducted using SPSS 17.0. Effect size (partial-eta squared) and power (*β*) values for ANOVAs were calculated in SPSS, and Cohen's d and power for t-tests were calculated with G*Power (Version 3.0.8).

## Results

### Hormone levels and physical measures

Serum testosterone levels were significantly lower in Antide-treated males (mean ± SEM: 0.60 ± 0.04 ng/ml) than control males (1.82 ± 0.42 ng/ml; *t*_22_ = 5.00, *p* < .001, *d* = 2.04, *β* = 1.00). Serum progesterone levels were significantly lower in Antide-treated females (0.58 ± 0.23 ng/ml) than control females (12.30 ± 2.76 ng/ml; *t*_22_ = 6.15, *p* < .001, *d* = 2.51, *β* = 1.00). Testes weights were significantly lower in Antide-treated males (0.22 ± 0.05 g) than control males (1.24 ± 0.04 g; *t*_22_ = 15.44, *p* < .001, *d* = 6.30, *β* = 1.00). Uterine weights were significantly lower in Antide-treated females (0.09 ± 0.02 g) than in control females (0.21 ± 0.03 g; *t*_22_ = 2.95, *p* = .007, *d* = 1.20, *β* = .80).

The interaction between sex, age and treatment group for ano-genital distances was significant (*F*_3, 132_ = 9.15, *p* < .001, *η*_*P*_^2^ = .17, *β* = 1.00): Antide-treated males had smaller ano-genital distances at pnd 35 (19.6 ± 3.3 mm) and pnd 40 (22.5 ± 0.5 mm) than same-aged control males (pnd 35 = 23.4 ± 0.8 mm: *p* < .001; pnd 40 = 27.4 ± 0.6 mm: *p* < .001), but not at pnd 21 or 28 (data not shown; pnd 21: *p* = .866; pnd 28: *p* = .158). Antide-treated females and control females did not differ in ano-genital distance at any age (data not shown; pnd 21: *p* = .931; pnd 28: *p* = .718; pnd 35: *p* = .586; pnd 40: *p* = .995).

For body weight, there was a significant interaction between sex, age and treatment group (*F*_3, 132_ = 11.26, *p* < .001, *η*_*P*_^2^ = .20, *β* = 1.00; Table 1). Control males were heavier than Antide-treated males at pnd 40 (*p* = .034) only (pnd 21: *p* = .982; pnd 28: *p* = .840; pnd 35: *p* = .435). Antide-treated females did not differ from control females at pnd 40 (*p* = .103) or other ages (pnd 21: *p* = .727; pnd 28: *p* = .978; pnd 35: *p* = .511). Control males were heavier than control females at pnd 35 (*p* = .006) and pnd 40 (*p* < .001) only (pnd 21: *p* = .586; pnd 28: *p* = .343).

### Time spent moving

There was a significant increase in the time spent moving from Trial 1 to Trial 2 (*F*_1, 44_ = 45.68, *p* < .001, *η*_*P*_^2^ = .51, *β* = 1.00; Table 2), with all subjects spending almost twice as long moving in Trial 2 compared to Trial 1. The main effects of sex and treatment group were non-significant (sex: *F*_1, 44_ = .13, *p* = .725; treatment group: *F*_1, 44_ = .57, *p* = .456). Whilst the Antide-treated subjects appeared to spent less time moving than the control subject in Trial 2 (*F*_1, 44_ = 5.91, *p* = .019, *η*_*P*_^2^ = .12, *β* = .66), the treatment group by trial interaction was not significant (*F*_1, 44_ = .06, *p* = .815), and no other interactions were significant (treatment group and sex: *F*_1, 44_ = .05, *p* = .830; trial and sex: *F*_1, 44_ = .03, *p* = .868).

### Total amount of contact with objects

Total amount of time spent in contact with the objects did not differ between trials (*F*_1, 44_ = .07, *p* = .796; Table 2), and there were no significant main effects of sex (*F*_1, 44_ = .12, *p* = .736) or treatment group (*F*_1, 44_ = .03, *p* = .871). All interactions were also non-significant (treatment group and trial: *F*_1, 44_ = .50, *p* = .485; treatment group and sex: *F*_1, 44_ = .92, *p* = .343; trial and sex: *F*_1, 44_ = .26, *p* = .615). Subjects spent around 110–130 s interacting with objects during each 5-minute trial.

### Preference for novelty

**Fig. 2:**
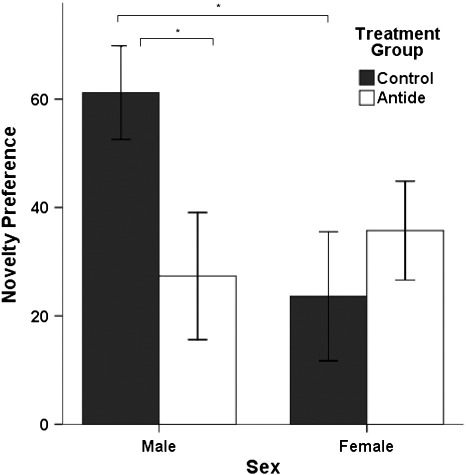
Mean preference for novelty during Trial 2 by sex and treatment group. Error bars represent ± 1 standard error of the mean. * *p* < 0.05 in post-hoc test.

A significant interaction between sex and treatment group was found for preference for novelty during Trial 2 (*F*_1, 44_ = 4.84, *p* = .033, *η*_*P*_^2^ = .10, *β* = .58; Fig. 2). Control males exhibited a stronger preference for the novel object than control females (*p* = .015), and Antide-treated males exhibited a significantly lower preference for the novel object than control males (*p* = .027), with a preference score similar to that of control females. Antide-treated females did not differ from control females in their preference for novelty (*p* = .416), and Antide-treated females exhibited similar scores to Antide-treated males (*p* = .573). Neither the main effect of treatment group nor sex was significant (treatment group: *F*_1, 44_ = 1.08, *p* = .305; sex: *F*_1, 44_ = 1.95, *p* = .169).

When the data for all subjects were combined, a one-sample *t*-test indicated that the animals showed a significant preference for the novel object over the familiar object (*t*_47_ = 6.74, *p* < .001, *d* = .97, *β* = 1.00), spending approximately 68.5% of the time with the novel object during the Trial 2. When each group was examined separately, a significant preference for the novel object was found for control males (*t*_11_ = 7.08, *p* < .001, *d* = 2.04, *β* = 1.00), Antide-treated males (*t*_11_ = 2.34, *p* = .039, *d* = .67, *β* = .57), and Antide-treated females (*t*_11_ = 3.92, *p* = .002, *d* = 1.13, *β* = .95), with a trend toward a preference in control females (*t*_11_ = 1.99, *p* = .073, *d* = .57, *β* = .44).

In order to test whether difference in preference for novelty between groups were related to differences in movement or total object contact, correlations between variables were examined. No significant relationships were found between preference for novelty and either movement duration or total object contact in Trial 1 (novelty preference and movement: *r*_48_ = −.03, *p* = .819; novelty preference and object contact: *r*_48_ = −.09, *p* = .539) or between novelty preference and movement duration or total object contact in Trial 2 (novelty preference and movement: *r*_48_ = .18, *p* = .220; novelty preference and object contact: *r*_48_ = .147, *p* = .320). In order to test whether a bias amongst the objects influenced preference for novelty, the analyses were repeated with object identity included as a random factor: the interaction between sex and treatment group remained significant (*F*_1, 40_ = 4.15, *p* = .048, *η*_*P*_^2^ = .094, *β* = .51), and the main effects of treatment group and sex remained non-significant (treatment group: *F*_1, 40_ = .59, *p* = .448; sex: *F*_1, 40_ = 1.36, *p* = .250).

## Discussion

This study examined the effects of suppressing gonadal hormone production in male and female rats on response to novel objects during adolescence. Subjects were treated with a GnRH antagonist, Antide, or a vehicle control from early adolescence (pnd 28) onwards and tested on the NOR task at pnd 40 (mid-adolescence). The results showed that Antide-treated male rats exhibited a lower preference for novelty compared to control males. In support of our previous research (Cyrenne and Brown, 2011), control females also exhibited a lower preference for novelty than control males at pnd 40. The hormone assays and physical measurements confirmed that treatment with the GnRH antagonist successfully suppressed gonadal hormone production in both males and females. The lack of significant effect of Antide treatment on preference for novelty in females indicates that the effect in males is not due to non-specific side effects of the drug treatment, and the lack of sex difference in locomotion supports our previous studies of adolescent behaviour in other novel environments (Lynn and Brown, 2009, 2010). Overall, the data suggest that the sex difference in response to novelty in adolescent rats is sensitive to manipulations of circulating testicular hormone levels.

Adolescent rodents have been characterised as showing higher novelty-seeking and risk-taking behaviour than adults, potentially due to selection pressures that have favoured a willingness to engage with novelty around the time of dispersal (e.g. Spear, 2000, 2007). However, relatively few studies of laboratory rodents have provided strong evidence in support of this characterisation (for an exception, see Douglas et al., 2003). Our study supports the suggestion that adolescent male rats have a strong preference for novelty when provided with a choice situation. Control males exhibited a stronger preference for novelty than females during mid-adolescence, and treatment of male rats with Antide specifically reduced preference when the subjects were given a ‘choice’ of interacting with a novel rather than a familiar object. Antide-treated males did not differ from control males in the total amount of time spent interacting with novel objects. The lack of drug treatment effect on total contact with objects is not obviously due to a floor effect, as the subjects in the current study spent a relatively large proportion of time interacting with objects (approximately 25 s/min), particularly when compared to previous NOR studies (e.g. 5 s/min; Aubele et al., 2008; Inagaki et al., 2010).

Suppression of gonadal hormone production in adolescent males could influence preference for novelty via a number of different mechanisms that can be broadly categorised as ‘memory capacity’ or ‘motivational’ explanations. Given that hippocampal lesions fail to influence performance on the NOR task when a short inter-trial interval is used (Ainge et al., 2006; Langston and Wood, 2010), the results of our study are not apparently due to the effects of gonadal hormone manipulation on hippocampal-dependent memory. Hormone manipulation could have instead influenced recognition memory processes that function relatively independently of the hippocampus (e.g. perirhinal cortex processing; Brown et al., 2010; Winters et al., 2008). However, Antide-treated males were able to distinguish between the novel and the familiar object, which suggests that recognition memory was at least not fully impaired. Alternatively, treatment of adolescent males with Antide could have influenced novelty preference per se, perhaps via interactions between testosterone and the dopamine system. Developmental changes in the dopaminergic neurotransmitter system have previously been suggested to play a role in the enhanced preference for novelty at adolescence (Doremus-Fitzwater et al., 2010), and the dopamine system is known to be responsive to gonadal hormones in adult rats (Becker, 1999; Gillies and McArthur, 2010). In addition, this system undergoes substantial developmental changes during adolescence (Andersen, 2003; Wahlstrom et al., 2010), and the developmental trajectories differ between males and females (e.g. Andersen and Teicher, 2000; Andersen et al., 1997). Given that memory processing and motivation systems are highly intertwined (Phillips et al., 2008; Wise, 2004), further investigations are required to uncover the exact neural mechanisms by which testicular hormones influence adolescent NOR performance.

Our finding that suppression of gonadal hormone production in adolescent male rats reduces preference for novelty supports a previous study on adult male rats. Aubele et al. (2008) reported that gonadectomy of adult males reduced response to novelty in an NOR task and that performance was restored by administration of testosterone propionate. As in our study, removal of testicular hormones did not affect the total amount of time spent interacting with objects, only the preference score in the final trial. Aubele et al. (2008) used a substantially longer inter-trial interval (1.5 h) than in the current study (2 min), so the proximate mechanisms underlying the two sets of results could differ greatly. Previous studies of adult female rats have also reported that gonadectomised subjects have a lower preference for the novel object in the NOR task than control subjects (Wallace et al., 2006) and that novelty preference is enhanced by administration of ovarian hormones (e.g. Frye and Walf, 2008; Frye et al., 2009; Inagaki et al., 2010; Jacome et al., 2010; Luine et al., 2003; Walf et al., 2006). These studies of adult females used a relatively long inter-trial interval (i.e. 4 h), whilst our study did not find a significant effects of gonadal hormone suppression on preference for novelty in adolescent females using a short inter-trial interval. The difference in results for adolescent and adult females could be due to the lower level of novelty preference shown by adolescent females compared to adults in the NOR paradigm (Cyrenne and Brown, 2011), which might have limited any effects of hormone suppression in our study due to a floor effect. An alternative possibility is that gonadal hormone manipulation influences NOR performance in adult female rats by affecting long-term memory processes, rather than by affecting novelty preference per se. From this perspective, Antide-treated adolescent females might have exhibited a reduced preference for the novel object at a longer inter-trial interval. Finally, manipulation of hormones in adolescent females might not influence response to novelty in the same manner as in adult females, as a result of developmental changes in relevant neural systems. Thus, whilst our results for female adolescents apparently contradict previous studies on adult females, several factors could explain the difference in findings.

Adolescence has been increasingly seen as a period of substantial development and sexual differentiation of the brain in rodents (Schwarz and McCarthy, 2008), and exposure to gonadal hormones during this period of life could have long-term impact on sex differences in brain function and behaviour (Becker, 2009; Doremus-Fitzwater et al., 2010; Ernst et al., 2009; Kuhn et al., 2010; Sato et al., 2008; Wahlstrom et al., 2010). Recent studies have provided strong experimental evidence that exposure to testicular hormones during adolescence has life-long effects on brain development and behaviour in rodents (e.g. Ahmed et al., 2008; Romeo et al., 2003; Schulz et al., 2003, 2004, 2006; reviewed by Schulz et al., 2009; Sisk and Zehr, 2005). Such research could be extended to investigate whether exposure to testicular hormones during adolescence has long-term impact on neural mechanisms underlying response to novelty.

## Figures and Tables

**Table 1:** Body weights at postnatal days 21, 28, 35 and 40 (grammes; means ± SEMs).

	Pnd 21	Pnd 28	Pnd 35	Pnd 40
Control males	33.9 ± 1.4	60.0 ± 2.2	110.4 ± 3.6^a^	136.4 ± 4.5^a^
Antide-treated males	34.0 ± 1.7	59.5 ± 2.2	97.3 ± 2.7	125.4 ± 4.0^b^
Control females	32.9 ± 1.1	57.4 ± 1.8	89.2 ± 2.3	111.6 ± 2.5
Antide-treated females	32.3 ± .9	57.4 ± 1.5	91.8 ± 2.3	119.9 ± 2.8

a = different from opposite-sex controls at *p* < 0.05.

b = different from same-sex controls at *p* < 0.05.

**Table 2:** Time spent moving (seconds; means ± SEMs) and total object contact during Trials 1 and 2 (seconds; means ± SEMs).

	Time spent moving		Total object contact	
Trial 1	Trial 2	Trial 1	Trial 2
Control males	103.9 ± 24.0	193.6 ± 5.6	124.1 ± 13.2	122.3 ± 11.3
Antide-treated males	97.6 ± 25.2	175.8 ± 6.0	108.4 ± 15.3	120.0 ± 16.1
Control females	110.5 ± 22.7	190.4 ± 4.0	118.2 ± 14.2	114.3 ± 8.0
Antide-treated females	103.8 ± 24.1	183.7 ± 4.2	128.5 ± 10.2	129.3 ± 13.6
